# Changes in School-Based Physical Activity and Well-Being Among Adolescents Before and After the COVID-19 Pandemic

**DOI:** 10.3390/healthcare14070836

**Published:** 2026-03-25

**Authors:** Dorota Groffik, Karel Frömel, Mateusz Ziemba

**Affiliations:** 1 Institute of Sport Sciences, Academy of Physical Education in Katowice, Mikołowska 72a, 40-065 Katowice, Poland; karel.fromel@upol.cz; 2 Faculty of Physical Culture, Palacký University Olomouc, třída Míru 117, 771 11 Olomouc, Czech Republic; 3 Chorzow Faculty, WSB Merito University in Poznań, Sportowa 29, 41-506 Chorzów, Poland; mateusz.ziemba@chorzow.merito.pl

**Keywords:** physical activity, physical education, well-being, active transport, COVID-19 pandemic

## Abstract

**Background:** To mitigate the negative impacts of the pandemic, it is essential to understand how the associations between different types of physical activity (PA) and adolescent well-being changed before and after the COVID-19 pandemic (defined here as the period marked by students’ return to stable in-person education). This study aimed to examine gender differences in the associations between school-related PA and subjective well-being before and after the pandemic. **Methods:** A cross-sectional design was used, including 430 boys and 571 girls from 22 high schools. Participants completed the Youth Activity Profile questionnaire to assess school-related and school-associated PA and the WHO-5 Well-Being Index to evaluate subjective well-being. Differences in participants’ PA across segments of the school day before and after the pandemic were evaluated using the Kruskal–Wallis test, and compliance with PA recommendations was analyzed using cross-tabulation and Pearson’s chi-square tests. **Results:** After the pandemic, both boys and girls reported significantly lower levels of active transportation to and from school compared with the pre-pandemic period. In addition, well-being levels were significantly lower in both genders after the pandemic. Before the pandemic, boys and girls with higher well-being met the recommendations for PA to school, from school, and outside of school significantly more often than their peers with lower well-being. Higher levels of well-being were observed both before and after the pandemic in boys and girls who participated in organized PA compared with non-participants. **Conclusions:** This study confirms lower levels of PA and well-being among adolescents after the pandemic. In particular, PA to and from school was at a lower level after the pandemic than before the pandemic. Participation in organized PA was significantly associated with higher well-being in both boys and girls before and after the pandemic. Supporting adolescents’ participation in organized PA should be a priority when addressing the negative consequences of societal crisis situations. Improved knowledge of the associations between PA and well-being may contribute to more effective support for adolescents’ PA and greater awareness of the importance of meeting PA recommendations.

## 1. Introduction

Insufficient physical activity (PA) remains a major global public health concern. Among adults worldwide, the prevalence of insufficient PA between 2000 and 2022 is alarming, and the global target of a 15% relative reduction by 2030 is unlikely to be achieved [1]. The situation is even more critical in adolescents, as approximately 81% of boys and girls fail to meet current global PA recommendations [2]. Globally, only 27–33% of children and adolescents achieve the recommended levels of moderate-to-vigorous PA (MVPA) [3], and at least two-thirds of European children and adolescents are insufficiently physically active [4]. The already unfavorable trends in adolescent PA were further exacerbated by the COVID-19 pandemic (hereafter referred to as the pandemic). A decline in PA has been documented in most countries worldwide as a result of pandemic-related restrictions [5,6,7,8], including a substantial decrease in PA among European children and adolescents [9].

In Polish and Czech schools, distance education during the pandemic was associated with a significant reduction in school-based PA, vigorous PA, and total PA in both boys and girls [10]. In addition, the proportion of Polish adolescents meeting the recommendations for MVPA declined markedly during the pandemic [11], accompanied by a deterioration in overall physical fitness [12].

Beyond its impact on PA, the pandemic also had profound consequences for adolescent mental health. Numerous studies have documented a deterioration in psychological well-being and an increase in mental health problems, including symptoms of stress, depression, anxiety, and sleep disturbances [13,14,15,16]. Globally, decreases in PA levels during the pandemic have been associated with lower well-being and increased anxiety and depressive symptoms among adolescents [17]. Similar negative trends in mental health were observed in Czech and Polish adolescents, particularly in the form of a marked increase in depressive symptoms. During the pandemic, only 35% of girls and 50% of boys reported high levels of well-being [18], followed by a subsequent decline in life satisfaction among Polish adolescents [19]. Many studies have confirmed significant associations between PA levels and well-being during the pandemic [20,21], suggesting that regular PA may play a protective role for mental health under conditions of self-isolation and social distancing [22]. Conversely, reductions in PA combined with increased sedentary screen time were associated with lower life satisfaction and greater psychological deprivation in adolescents [23]. These findings highlight that health-promoting interventions targeting physically inactive adolescents may be particularly important for improving well-being [24].

The school environment plays an irreplaceable role in mitigating the negative effects of pandemic-related restrictions on adolescents’ physical and psychological health [10]. However, schools often do not fully exploit their potential to support PA and mental well-being [25], even if positive associations between the school environment and adolescent PA are reported [26]. Opportunities provided by physical education (PE) lessons and physically active school breaks [27], as well as comprehensive school-based PA programs [28], remain underutilized. School PA is inversely associated with mental ill health [29], but the quality of evidence on positive associations between PA and the mental health of children and adolescents is still insufficient [30]. Comparisons in associations between types of PA and adolescents’ mental health in the periods before and after the pandemic have not yet been extensively researched. Changes in types of PA before and after the pandemic in Czech and Polish schools were analyzed in previous research [31]. A deeper understanding of the associations between different types of school-based and school-related PA and adolescents’ well-being, examined across distinct periods before and after the pandemic, may contribute to more effective promotion of physically active and healthy lifestyles among adolescents.

Therefore, the aim of this study was to determine gender differences in the associations between types of school-related PA and well-being before and after the pandemic. The following research questions were addressed:

- Which types of school-related PA are associated with higher well-being?
- What gender differences exist in the associations between PA types and well-being?
- What gender differences exist in the fulfillment of PA recommendations across PA types?
- What are the associations between participation in organized PA and adolescents’ well-being?

## 2. Methods

### 2.1. Study Design and Setting

A cross-sectional study was conducted during two periods: in the two years before the pandemic and in the two years after the pandemic. The post-pandemic period could not be determined by a specific date due to significant differences across schools and the ongoing negative impacts of the pandemic. The decisive factor was the return of all students to face-to-face education, which marked the beginning of the post-pandemic period. Before the pandemic, data were collected in 13 high schools, while after the pandemic, data were obtained from 9 high schools. Schools with long-term cooperation with the Jerzy Kukuczka Academy of Physical Education in Katowice were invited to participate. Before the pandemic, only schools in which the same research had not been conducted in previous years were selected. After the pandemic, it was not possible to recruit the same schools as before the pandemic due to organizational constraints. This is a stratified and quota-based set of schools and groups of students selected for purpose. Groups of students in schools were randomly selected. The situation at schools after the pandemic was very demanding, and school management could not afford to burden students with additional tasks related to research. Therefore, schools of comparable type, size and location in the same regions were selected to ensure the greatest possible comparability between the two periods. Before the pandemic, almost all schools agreed to participate in the research, whereas after the pandemic almost 50% declined participation with an apology. Data collection was carried out using the web-based application International Database for Research and Educational Support (Indares). School management was informed about the anonymization of data through individual participant codes and about the use of aggregated group results for feedback purposes.

### 2.2. Participants

In each participating school, two classes from the same year were randomly selected, ensuring that students who had previously completed similar research were not included in the study. In total, 430 boys and 571 girls participated in the research. All participants and their parents provided written informed consent prior to participation. Participants could withdraw from the study at any time during the data collection process; however, all students completed the research procedures. A total of 33 boys and 21 girls were excluded due to failure to meet the data processing criteria in the individual questionnaires. Participants were usually informed about the average results of the research within three weeks after the completion of data collection.

### 2.3. Procedures and Measurements

The initial meeting with participants was conducted in school computer laboratories. School-based and school-related PA was assessed using the Youth Activity Profile (YAP) questionnaire [32]. The questionnaire was translated following the guidelines of the EORTC Quality of Life Group [33]. Standardization of the questionnaire was conducted for the Czech version only, using accelerometer-based monitoring with ActiGraph devices (GT9X LINK and wGT3X+; ActiGraph Corp., Pensacola, FL, USA). The validity coefficients for the summary outcomes ranged from r_s_ = 0.40 to 0.49 [34]. At present, the calibrated algorithm for estimating moderate-to-vigorous physical activity cannot be fully applied in the Polish context [35,36]. The YAP questionnaire was administered electronically via the Indares web application.

The YAP questionnaire uses a five-point Likert scale and includes two items assessing PA during travel to and from school (number of days per week), three items assessing PA during school time (PE lessons, school breaks or study hall, and lunch breaks; use of time for PA), and three items assessing PA outside of school lasting at least 10 min (before school, after school, and in the evening; number of days per week). In the present study, PA after school and evening PA were combined into a single indicator of after-school PA.

Physical activity recommendations were defined as follows: at least one day per week of PA before school; at least four days per week of PA to school, from school, and after school. For school breaks and lunch breaks, average use of time for PA was evaluated, while for PE lessons, use of a large proportion of lesson time for PA was considered [31]. The analyses focused on school-based PA and school-related PA only. Physical activity was evaluated for individual segments of the school day, as well as for aggregated indicators: school-based PA (PE lessons, breaks, and lunch breaks), outside-of-school PA (to and from school, before and after school), and total PA across the school day. Results are presented as summed scores based on the Likert scale.

Subjective well-being and symptoms of depression were assessed using the WHO-5 Well-Being Index (Polish version, https://www.psykiatri-regionh.dk/who-5/Pages/default.aspx, accessed on 15 February 1019) [37,38], which evaluates well-being during the preceding two weeks. The WHO-5 is a validated and widely used tool for group comparisons in population-based research [39]. Participants were stratified into a lower well-being group (<13 points) and a higher well-being group (≥13 points).

### 2.4. Statistical Analysis

Data were analyzed using Statistica software, version 14.0.0.15 (StatSoft, Prague, Czech Republic). Descriptive statistics were used to characterize the study sample. The Kolmogorov–Smirnov and Lilliefors tests were applied to assess data normality. Differences in PA across segments of the school day before and after the pandemic were evaluated using the Kruskal–Wallis test. Differences in the achievement of PA recommendations were analyzed using cross-tabulation and Pearson’s chi-square tests. Effect sizes were expressed using eta squared (η^2^) and the correlation coefficient (r), interpreted as follows: η^2^ = 0.01–0.05 (r = 0.10–0.29), small effect; η^2^ = 0.06–0.13 (r = 0.30–0.49), medium effect; and η^2^ ≥ 0.14 (r ≥ 0.50), large effect [40,41]. The level of statistical significance was set at *p* < 0.05.

## 3. Results

### 3.1. Sample Characteristic

In total, 1001 students participated in the research. At each participating school, two classes from the same grade were randomly selected. Before the pandemic, the sample included 306 boys (mean age = 15.0 ± 1.7 years; mean body mass = 63.0 ± 14.2 kg; mean height = 171.2 ± 11.4 cm; mean BMI = 21.3 ± 3.2 kg/m^2^) and 338 girls (mean age = 15.3 ± 1.6 years; mean body mass = 54.6 ± 9.8 kg; mean height = 162.8 ± 7.0 cm; mean BMI = 20.5 ± 3.1 kg/m^2^). After the pandemic, the sample consisted of 124 boys (mean age = 15.8 ± 0.7 years; mean body mass = 65.4 ± 11.1 kg; mean height = 177.5 ± 7.5 cm; mean BMI = 20.7 ± 2.9 kg/m^2^) and 233 girls (mean age = 15.6 ± 0.8 years; mean body mass = 55.9 ± 8.5 kg; mean height = 165.9 ± 5.9 cm; mean BMI = 20.3 ± 2.7 kg/m^2^).

Before the pandemic, participation rates exceeded 80% in most schools, whereas after the pandemic, the average participation rate decreased to approximately 70%. Before the pandemic, 65% of boys and 57% of girls participated in organized PA, including sports training. After the pandemic, participation in organized PA was reported by 53% of boys and 55% of girls.

### 3.2. Differences in the PA of Boys and Girls in the Segments of the School Day Before and After the Pandemic

After the pandemic, significantly lower levels of active transportation to school were observed in both boys (*p* < 0.001) and girls (*p* = 0.001) compared with the pre-pandemic period. Similarly, active transportation from school decreased significantly after the pandemic in boys (*p* = 0.003) and girls (*p* = 0.004) (Table 1). In boys, significantly lower levels of PA were also found after the pandemic in the after-school segment (*p* = 0.049), in the evening (*p* = 0.010), during school breaks (*p* = 0.021), and during lunch breaks (*p* < 0.001) compared with the pre-pandemic period. No significant differences were observed between boys and girls in any type of school-based or school-related PA after the pandemic.

### 3.3. Differences in the Well-Being of Boys and Girls Before and After the Pandemic

A significantly lower level of well-being after the pandemic was observed in both boys and girls for several WHO-5 items (Table 2). Boys showed significantly lower levels on item 1 (“I felt cheerful and in a good mood”; *p* = 0.043), item 3 (“I felt active and vital”; *p* < 0.001) and in the overall well-being score (*p* < 0.001). Girls showed significantly lower levels on item 1 (*p* = 0.002), item 2 (“I felt calm and relaxed”; *p* < 0.001), item 3 (*p* = 0.003,) and the overall well-being score (*p* = 0.016). Before the pandemic, boys reported significantly higher overall well-being than girls (*p* < 0.001), with a mean score of 15.5 points compared with 12.4 points in girls. This gender difference remained significant after the pandemic (*p* < 0.001), with boys reporting a mean score of 13.9 and girls 11.1.

### 3.4. Achievement of Physical Activity Recommendations in Segments of the School Day Boys and Girls Before and After the Pandemic

The most pronounced differences in the achievement of PA recommendations before and after the pandemic were observed in active transportation to and from school in both boys and girls (Table 3). In both genders, the proportion of adolescents meeting the recommendation for PA to and from school was significantly lower after the pandemic compared with before the pandemic. In contrast, girls showed a significantly higher proportion meeting the recommendation for PA during PE after the pandemic compared with before the pandemic (*p* = 0.005). No other significant changes were observed in the achievement of PA recommendations for after-school or evening PA.

### 3.5. Differences in the Achievement of PA Recommendations in Boys and Girls with Lower and Higher Well-Being

**Figure 1:**
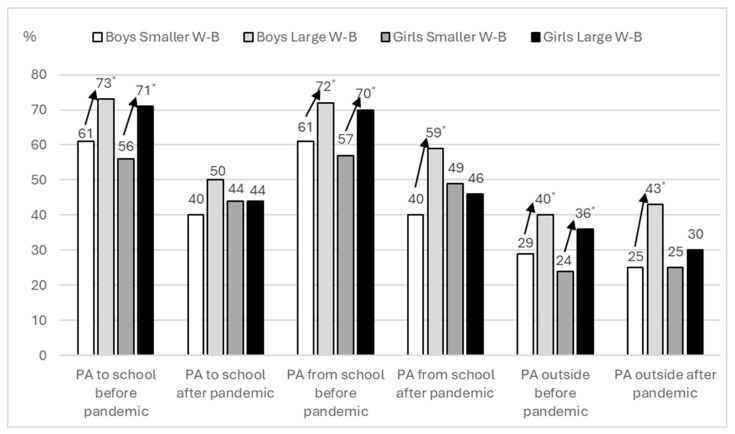
Differences in the achievement of recommendations for physical activity (PA) to school, from school and outside school in boys and girls with lower and higher well-being (W-B) before and after pandemic (* *p* < 0.05).

Before the pandemic, both boys and girls with higher levels of well-being met the recommendations for PA to school, from school, and outside of school significantly more often than their peers with lower well-being (Figure 1). After the pandemic, this pattern was largely attenuated. Only boys with higher well-being met the recommendation for PA from school and for outside-of-school PA significantly more often than boys with lower well-being. No significant differences were observed between well-being groups in girls after the pandemic.

### 3.6. Well-Being of Boys and Girls Before and After Pandemic According to Participation in Organized Physical Activity

**Figure 2:**
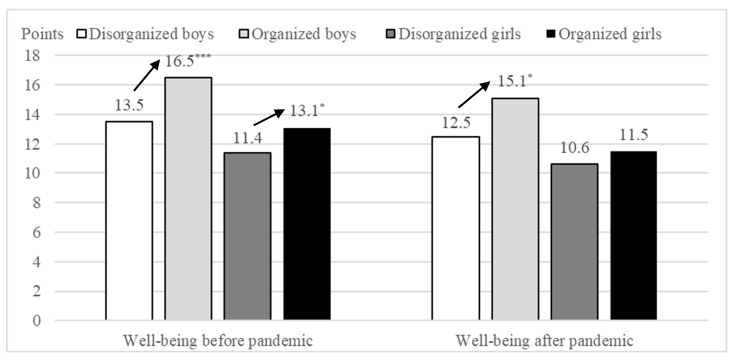
Well-being of boys and girls before and after pandemic according to participation in organized physical activity (* *p* < 0.05; *** *p* < 0.001).

Participation in organized PA was significantly associated with higher well-being in both boys and girls before and after the pandemic (Figure 2). In boys, significant differences in well-being were observed between participants and non-participants both before (H_(3,430)_ = 44.16, *p* < 0.001, η^2^ = 0.097) and after (*p* = 0.022) the pandemic. In girls, participants in organized PA reported significantly higher well-being than non-participants before the pandemic (*p* = 0.011), whereas no significant difference was observed after the pandemic.

When interpreting the results of school PA for boys and girls, it is important to consider gender differences in relation to PA and PE. A significant finding is that 63% of boys (44% of girls) reported a positive relationship to PA before the pandemic, while only 51% of boys (36% of girls) reported a positive relationship to PA after the pandemic. It is very sad that 56% of boys (27% of girls) reported a positive relationship to PE before the pandemic, but only 36% of boys (15% of girls) reported a positive relationship to PE after the pandemic.

## 4. Discussion

The main finding of this study is that after the pandemic the participants showed a lower levels of active transport to and from school than participants attending schools before the pandemic. Moreover, no significant gender differences were observed in any type of school-based or school-related PA after the pandemic. Similar patterns have been reported in children, where PA levels did not return to pre-pandemic values after the relaxation of restrictive measures, despite parental PA returning to pre-pandemic levels [42]. Parental and social support appear to play an important role in mitigating unfavorable PA behaviors, as social support has been shown to be significantly associated with PA levels [43].

After the pandemic, a decrease in PA was also reported among Danish adolescents [44]. In contrast, Hurter et al. [45] documented an increase in adolescent PA following the return to school; however, their findings were based on comparisons with PA levels during the pandemic rather than with pre-pandemic values. These differences highlight the importance of carefully interpreting post-pandemic PA data in relation to the specific reference period used.

Gender differences in PA patterns after the pandemic appear to be influenced primarily by a greater decline in PA among boys during the pandemic compared with girls [46,47]. One potential explanation is the reduced participation of adolescents in organized PAy during and after the pandemic [48,49]. Participation in organized PA plays a crucial role in maintaining an active lifestyle, including in the Polish context [50]. Ensuring equitable access to organized PA has therefore become an increasingly important priority in after the pandemic [51]. There is growing consensus that pandemic-related restrictions may accelerate long-term declines in PA among children and adolescents, with potential lasting consequences for physical health [52], mental health [53], and broader demographic development [54]. Moreover, the high prevalence of physical inactivity may prove difficult to reverse in the coming years [6].

Physical activity levels immediately after the return to school following the pandemic should be interpreted with caution, particularly given the reliance on self-reported measures. The widespread use of wearable devices for PA monitoring was not feasible in the immediate post-pandemic school environment. Nevertheless, the validity of subjective assessments of PA is supported by findings from Cocca et al. [55], who reported strong correlations between self-reported PA and accelerometer-based measurements (r = 0.767–0.968).

In this study, significantly lower well-being was observed in both boys and girls after the pandemic, with boys consistently reporting higher well-being than girls both before and after the pandemic. A greater negative impact of the pandemic on girls’ well-being has also been documented in adolescents from Iceland [56], Norway [57], Japan [58], and in Czech and Polish populations [10]. Although positive associations between specific types of PA (such as active transportation and PA outside school before school) and well-being were evident before the pandemic, these associations were not confirmed after the pandemic. Nevertheless, the overall pattern indicates that lower PA levels are associated with lower well-being, reinforcing the importance of PA as a determinant of adolescent mental health [59].

These findings are consistent with Canadian research showing a greater decline in PA during the pandemic among less physically active individuals compared with their more active peers. Importantly, among inactive adolescents, significant differences in well-being were observed between those who were more physically active and those who were less active, whereas such differences were not evident among physically active adolescents [24]. In addition to activity type, PA intensity appears to be an important factor. Vigorous PA has been associated with positive effects on well-being [60], although these associations may be moderated by contextual and individual factors, and negative effects on subjective well-being have also been reported [61]. Physical activity frequency also plays a role, with more frequent activity being linearly associated with better self-rated well-being in adults [62]. Similarly, among European adolescents, higher PA frequency and participation in sport have been independently associated with greater well-being and lower levels of anxiety and depressive symptoms in both boys and girls [63]. Interventions that strengthen the association between PA and core self-evaluation also appear to be important for supporting adolescent well-being [64].

The associations observed between PA types and well-being before and after the pandemic correspond closely with patterns in the fulfillment of PA recommendations. Before the pandemic, boys and girls with higher well-being met PA recommendations significantly more often than their peers with lower well-being. After the pandemic, however, these differences were largely attenuated, with significant associations observed only in boys for PA from school. The lack of consistent post-pandemic associations between meeting PA recommendations and well-being suggests that the relationship between PA and mental health may have been modified by broader psychosocial consequences of the pandemic. Further longitudinal research is needed to clarify these relationships in the post-pandemic context.

Finally, the present findings highlight the important role of participation in organized PA in supporting adolescent well-being. Boys and girls who participated in organized PA demonstrated higher levels of well-being both before and, to a lesser extent, after the pandemic. This finding underscores the potential of organized PA as a protective factor during societal crisis situations, including pandemics and other large-scale disruptions.

### Strength and Limitations

This study offers a novel approach to the assessment of school-based and school-related PA by examining different types of PA across distinct periods before and after the pandemic using the Youth Activity Profile questionnaire. An additional strength is the application of newly defined PA recommendations for specific segments of the school day, which allows for a more nuanced evaluation of adolescents’ PA patterns in the school context.

Several limitations should be acknowledged. The study samples before and after the pandemic were purposefully selected, as it was not feasible to conduct the research on representative samples during both periods. Due to organizational and educational constraints following the pandemic, it was not possible to involve the same schools after the pandemic data collection as before the pandemic. Unfortunately, in the difficult period after the pandemic, it was not possible to monitor PA using wearable devices. Although schools of comparable type, size, and region were selected, this limits direct comparability between the two samples. Consequently, the findings allow for the identification of differences in PA and well-being before and after the pandemic but do not permit causal inferences or definitive conclusions regarding changes over time. Therefore, significant differences in the results before and after the pandemic should be approached with caution, also due to the low coefficient of the effect size. This limitation also restricts the potential for direct replication of the study design. Nevertheless, future studies conducted in similar contexts may corroborate the observed patterns and further validate the study’s conclusions.

## 5. Conclusions

This study is among the first to examine associations between different types of PA and subjective well-being in adolescent boys and girls after the pandemic, while explicitly contextualizing these associations with pre-pandemic PA patterns. Immediately after the pandemic restrictions were lifted, lower levels of PA and subjective well-being were observed for both boys and girls compared to results found at similar types of schools before the pandemic. Gender differences in PA and subjective well-being were not significant after the pandemic. In the post-pandemic school environment, a key priority should be the systematic support of school-related PA, alongside increased support for adolescents’ participation in organized PA. This is particularly important for less physically active adolescents and for girls, who consistently report lower levels of PA and well-being. Regular monitoring of school-based PA, school-related PA, and participation in organized PA may contribute to reducing gender disparities and promoting healthier lifestyles among adolescents. Expanding access to organized PA through school-based extracurricular programs and community sports facilities represents an important public health strategy for mitigating the long-term negative consequences of pandemic-related disruptions on adolescent physical and mental health.

## Figures and Tables

**Table 1:** Physical activity of boys and girls in segments of the school day before and after pandemic.

Physical Activity	Boys				Girls				H	*p*	η^2^
	Before (n = 306)		After (n = 124)		Before (n = 338)		After (n = 233)				
	M	SD	M	SD	M	SD	M	SD			
	**Days**										
Before school	2.20	1.50	1.66	1.20	0.81	1.28	0.71	1.30	25.50 ^a^	<0.001	0.023 *
To school	2.95	1.62	2.05	1.88	2.79	1.66	2.18	1.81	43.34 ^a,b^	<0.001	0.040 *
From school	2.99	1.59	2.24	1.85	2.86	1.62	2.46	1.74	26.58 ^a,b^	<0.001	0.024 *
After school	2.29	1.43	1.87	1.49	1.88	1.47	1.77	1.45	20.99 ^a^	<0.001	0.018 *
Evenings	2.12	1.50	1.85	1.54	1.74	1.46	1.71	1.44	13.49 ^a^	0.004	0.011 *
	**Time (average use of the time for physical activity)**										
During PE	4.07	1.17	3.80	1.52	3.43	1.43	3.67	1.20	47.34	<0.001	0.044 *
During breaks	2.38	1.46	1.89	1.20	1.80	1.26	1.80	1.19	34.94 ^a^	<0.001	0.032 *
During lunch breaks	2.10	1.59	1.47	1.48	1.61	1.43	1.31	1.29	38.35 ^a^	<0.001	0.035 *

M: mean; SD: standard deviation; H: Kruskal–Wallis test; *p*: significance level; η^2^: effect size coefficient; * small effect size; ^a^ Boys–Significant difference between before and after pandemic; ^b^ Girls–Significant difference between before and after pandemic. PE—physical education lessons; Days—number of school days with physical activity; Time—the number of points evaluating the use of time of physical activity (from 0 points—I didn’t have physical education to 5 points—almost all of the time).

**Table 2:** Differences in the level of well-being of boys and girls before and after the pandemic.

Statements	Boys				Girls				H	*p*	η^2^
	Before (n = 306)		After (n = 124)		Before (n = 338)		After (n = 233)				
	M	SD	M	SD	M	SD	M	SD			
1. I have felt cheerful and in good spirits	3.51	1.14	3.16	1.14	3.10	1.27	2.76	1.14	57.05 ^a,b^	<0.001	0.054 *
2. I have felt calm and relaxed	3.08	1.27	2.84	1.20	2.38	1.35	2.03	1.21	92.93 ^b^	<0.001	0.090 **
3. I have felt active and vigorous	3.45	1.28	2.84	1.27	2.68	1.50	2.30	1.26	97.90 ^a,b^	<0.001	0.095 **
4. I woke up feeling fresh and rested	2.26	1.56	2.10	1.43	1.61	1.45	1.50	1.38	48.64	<0.001	0.046 *
5. My daily life has been filled with things that interest me	3.17	1.43	2.95	1.40	2.61	1.56	2.50	1.37	34.96	<0.001	0.032 *
Summary raw score	15.47	4.76	13.90	4.87	12.38	5.62	11.09	4.82	103.43 ^a,b^	<0.001	0.101 *

M: mean; SD: standard deviation; H: Kruskal–Wallis test; *p*: significance level; η^2^: effect size coefficient; * small effect size; ** medium effect size; ^a^ Boys—Significant difference between before and after pandemic; ^b^ Girls—Significant difference between before and after pandemic.

**Table 3:** Achievement of physical activity recommendations across segments of the school day in boys and girls before and after the pandemic.

Physical Activity Levels	Boys					Girls				
	Before %	After %	χ^2^	*p*	r	Before %	After %	χ^2^	*p*	r
**Days**										
Before school (1 day)	47	30	10.74	0.001	0.158	36	29	2.85	0.091	0.071
To school (≥4 days)	67	44	18.92	<0.001	0.210	61	44	15.01	<0.001	0.162 *
From school (≥4 days)	67	48	12.44	<0.001	0.170	61	48	9.71	0.002	0.130 *
After school or evenings (≥4 days)	35	33	0.10	0.755	0.015	28	26	0.26	0.612	0.021
**Time (use of the time for physical activity)**										
During PE lessons (lot of the time)	76	73	0.72	0.397	0.041	57	69	8.03	0.005	0.119 *
During breaks (moderate/average)	46	31	8.30	0.004	0.139	28	27	0.01	0.928	0.004
During lunch breaks (moderate/average)	41	24	10.62	0.001	0.157	28	19	6.37	0.012	0.106 *

M: mean; SD: standard deviation; χ^2^: Pearson’s chi-square; *p*: significance, r: effect size coefficient; * small effect size; PE: physical education; Days: number of school days with physical activity; Time: the number of points evaluating the use of time of physical activity (from 0 points—I didn’t have physical education to 5 points—almost all of the time).

## Data Availability

The data presented in this study are available on request from the corresponding author due to privacy or ethical restrictions.
